# Dietary *Tenebrio molitor* Larvae Meal Inclusion Exerts Tissue-Specific Effects on Cellular, Metabolic, and Antioxidant Status in European Sea Bass (*Dicentrarchus labrax*) and Gilthead Seabream (*Sparus aurata*)

**DOI:** 10.1155/2022/9858983

**Published:** 2022-10-20

**Authors:** Thomas Bousdras, Konstantinos Feidantsis, Nikolas Panteli, Stavros Chatzifotis, Giovanni Piccolo, Laura Gasco, Francesco Gai, Efthimia Antonopoulou

**Affiliations:** ^1^Laboratory of Animal Physiology, Department of Zoology, School of Biology, Aristotle University of Thessaloniki, GR-54124 Thessaloniki, Greece; ^2^Institute of Marine Biology, Biotechnology and Aquaculture, Hellenic Centre for Marine Research, Gournes Pediados, P.O. Box 2214, GR-71003, Heraklion, Crete, Greece; ^3^Department of Veterinary Medicine and Animal Production, University of Naples Federico II, Via F. Delpino 1, 80137 Naples, Italy; ^4^Department of Agricultural, Forest and Food Sciences, University of Turin, Largo Paolo Braccini 2, 10095 Grugliasco, Italy; ^5^Institute of Sciences of Food Production, National Research Council, Largo Paolo Braccini 2, 10095 Grugliasco, Italy

## Abstract

The present study addresses the effects of dietary *Tenebrio molitor* (TM) larvae meal inclusion on cytoprotective, cell death pathways, antioxidant defence, and intermediate metabolism in the heart, muscle, and digestive tract of gilthead seabream (*Sparus aurata*) and European sea bass (*Dicentrarchus labrax*). Three experimental diets were formulated to contain 0%, 25%, or 50% inclusion TM levels. Heat Shock Proteins (HSPs) induction was apparent in both species' muscle at 50% inclusion. Conversely, p44/42 Mitogen-Activated Protein Kinase (MAPK) activation was increased (*p* < 0.05) in both species' muscle and digestive tract at 25% inclusion. Regarding the apoptotic machinery, TM inclusion exerted no influence on gilthead seabream, while suppression through autophagy may have occurred in the muscle. However, significant apoptosis (*p* < 0.05) was evident in European sea bass muscle and digestive tract. Both fish species' heart seemed to additionally rely on lipids compared to muscle and digestive tract. In contrast to gilthead seabream, European sea bass exhibited increased (*p* < 0.05) antioxidant activity at 50% TM inclusion. The present findings highlight the dietary derived induction of cellular responses in a species- and tissue-specific manner, whereas European sea bass appears to be more susceptible to TM inclusion.

## 1. Introduction

Fish meal, the basal protein source in compound fish feed, is produced by captured wild fish populations, mainly small pelagic fish [1]. Integration of the aquaculture industry in the sustainable food systems premises potent changes in order to lessen the progressive depletion of wild fish stocks [2–6]. The latter, along with the sharp, increases in fish meal price [1], incentivize the seeking of nutritionally appropriate, and environmentally-sustainable alternatives to fish meal [7–9].

The recent authorization by the EU Commission (EU regulation 2017/893) for the utilization of processed animal protein, derived from seven insects' species in aquafeeds, reinforced the scientific inquiry regarding the impact of insect meal on fish. Several carnivorous and omnivorous fish species consume insects as a natural nutrient source (reviewed in [10]). In general, insects display a high crude protein level and a well-balanced profile of essential amino acids, while they are simultaneously good sources of energy, lipids, vitamins, and minerals [10–12]. In contrast to conventional livestock, industrial production of insects requires much less land, energy, and water resources and produces lower greenhouse gas and ammonia emissions [13, 14]. In addition, insects have high feed conversion efficiency due to their cold-blooded nature and can biodegrade and recycle organic and agricultural waste into high-quality feed [15, 16].

Among the authorized species, yellow mealworm, *Tenebrio molitor* (Linnaeus, 1758), has gained great attention, and its suitability as ingredient in fish feeds has been studied in relation to various aspects of several reared fish species, including European sea bass *Dicentrarchus labrax* (Linnaeus, 1758) [11, 17], rainbow trout *Oncorhynchus mykiss*(Walbaum, 1792) [18, 19], gilthead seabream *Sparus aurata* (Linnaeus, 1758) [20, 21], and European perch *Perca fluviatilis* (Linnaeus, 1758) [22]. Dietary inclusion of yellow mealworm has been reported to exert positive or no effect on fish growth performance, nutrient digestibility, and body composition, depending on the species [11, 19, 23]. However, high yellow mealworm inclusion levels may adversely affect growth rate and/or nutrient digestibility [21, 23], possibly due to the imbalanced amino acid profile [24]. Moreover, yellow mealworm consumption has been reported to showcase ameliorative effects on fish antibacterial and antiparasitical defence [17], gut microbiota [20, 25], and both innate and adapted immune response [26].

Nutrient requirements, digestion, and metabolic utilization differ among fish species depending on natural dietary habits and adaptations [27, 28]. Modification of diet composition may lead to nutrient deficiencies, especially in the case of formulated diets where nutrient bioavailability is highly sensitive to feedstuff processing techniques [28]. Dietary restrictions due to feed deprivation or changes in fish feed composition have been reported to induce oxidative stress and affect cellular mechanisms [29, 30]. In turn, oxidative stress can trigger protein oxidation/denaturation, lipid peroxidation, and apoptosis-mediated cell death, thus leading to physiological dysfunction [31, 32]. Apoptosis cleaves damaged cells without affecting tissue structure or function [33], when autophagy cannot sustain tissue homeostasis by the recycling of abnormal cytoplasmic organelles and malfunctioning proteins [34]. The mitogen-activated protein kinase (MAPK) pathway is activated by several stress stimuli and transduces extracellular signals to regulate cellular processes, including apoptosis [35]. Furthermore, requirement of Heat Shock Proteins (HSPs) for the prevention of protein unfolding during dietary-induced stress [36] has been previously reported in fish species [37, 38].

However, stress stimuli induce cellular response and apoptosis in a tissue-specific manner [39–41]. Thus, the present study aims to address the species- and tissue-specific effects of yellow mealworm inclusion on cellular responses, antioxidant defence, and apoptosis and autophagy pathways in European sea bass and gilthead seabream. Both gilthead seabream and European sea bass consist two of the most economically valuable teleost species for the commercial aquaculture especially in the Mediterranean region [42]. However, because these fish species could be subjected to adverse stimuli (including nutritional) for a prolonged period [43] and because their biology is different, their biochemical response to several versatile stimuli is of scientific interest and needs to be addressed.

## 2. Materials and Methods

### 2.1. Dietary Experiments and Sampling

Two independent dietary trials were conducted using two farmed fish species: gilthead seabream and European sea bass. The experiments are described in detail in Gasco et al. [44] for European sae bass and in Piccolo et al. [21] for gilthead seabream. As partial fish meal substitution, the same full-fat yellow mealworm (*T. Molitor*, TM) larvae meal, purchased from the Gaobeidian Shannong Biology CO. LTD (Shannong, China) (Italy), was used in the fish diets. Ingredients and proximate composition of experimental diets are reported in Table 1 [21, 44]. The TM compositions are the following: dry matter = 93.9%, ash (%as fed) = 4.7%, crude protein (%as fed) = 51.9%, ether extract (%as fed) = 23.6%, and gross energy (MJ/kg as fed) = 24.4, while the fatty acid profile is described in detail in Gasco et al. [44]. Formulated diets were designed to meet the different nutritional requirements of each fish species.

The experimental protocols were designed according to the guidelines of the current European Directive (2010/63/EU) on the protection of animals used for scientific purposes. The gilthead seabream trial was performed at the Department of Veterinary Medicine and Animal Production (University of Naples Federico II, Italy), as described in Piccolo et al. [21] and was approved by the Ethic Committee of Federico II University. The European sea bass trial was performed at the Institute of Marine Biology, Biotechnology and Aquaculture (IMBBC) of the Hellenic Center for Marine Research (Crete, Greece) (EL91- BIOexp-04), as described in Gasco et al. [44] and was approved by the Aquaexcel Ethic Committee (Ref 0013/03/05/15B and Ref. 0125/08/05/15/TNA).

Briefly, as described in Piccolo et al. [21], gilthead seabream [initial body weight 105.2 ± 0.17 g (mean ± SD)] and European sea bass juveniles [initial body weight 5.2 ± 0.82 g (mean ± SD)] were fed three isonitrogenous, isolipidic, and isoenergetic diets, for 163 and 70 days, respectively [44]. In specific, a control diet (TM0 - TM was included at a level of 0%) in which fish meal (FM) was the main protein source, and TM25 and TM50 diet in which 25% and 50% (well above the recommended level of 10%), respectively, of TM larvae meal was added to the diet as partial substitution of FM, as described in Antonopoulou et al. [25]. In both nutritional trials, fish were daily fed to apparent satiation.

At the end of each growth trial, following a fasting period of one day, five healthy fish from each dietary group were removed and sacrificed by anaesthesia overdose [tricaine methanesulfonate-MS222 [(400-500 mg l^−1^ of sea water) Sigma Aldrich, St. Louis, MO, USA] [45]. The fish body weight was measured. Thereafter, ice fish were dissected, and the muscle, heart, and digestive tract (intestine without pyloric caeca) tissues were sampled in vials and stored in liquid nitrogen. Samples were then transferred to the laboratory where they were kept in -80°C until further analysis.

### 2.2. Determination of HSPs, MAPKs, Apoptosis, Autophagy, and Ubiquitination Levels (Immunoblot Analysis)

The preparation of tissue samples for SDS-PAGE, quantification of caspases and ubiquitinated proteins, and the immunoblot analysis are based on well-established protocols. Specifically, for the SDS-PAGE in the present study, equivalent amounts of proteins (50 *μ*g), from muscle, heart, and digestive tract tissues of 5 individual animals from each species and diet regime, were separated either on 10% and 0.275% or 15% and 0,33% (w/v) acrylamide and bisacrylamide. Thereafter, they were electrophoretically transferred onto nitrocellulose membranes. Antibodies used were as follows: monoclonal mouse anti-heat shock protein 70 kDa (H5147, Sigma), monoclonal mouse anti-heat shock protein 90 kDa (H1775, Sigma), monoclonal mouse anti-phospho-SAPK-JNK (Thr183-Tyr185) (Cell Signaling), monoclonal rabbit anti-phospho p44/42 MAPK (Tgr202/Tyr204) (4376, Cell Signaling), polyclonal rabbit anti-phospho-p38 MAP kinase (Thr180-Tyr182) (9211, Cell Signaling), monoclonal rabbit anti-LC3B (3868, Cell Signaling), polyclonal rabbit anti-p62/SQSTM1 (5114, Cell Signaling), anti-Bcl2 (7973, Abcam), and anti-Bax (B-9) (2772, Cell Signaling). Quality transfer and protein loading, in both western blot and dot blot, were assured by Ponceau stain and actin (anti-*β* actin 3700, Cell Signaling). Quantification of caspases and ubiquitinated proteins was assessed in a solid- phase immunochemical assay. The antibodies used were a polyclonal anti-ubiquitin rabbit antibody (3936, Cell Signalling) and anti-cleaved caspase antibody (8698 Cell Signalling). After washing in TBST (3 periods, 5 min each time), the blots were incubated with horseradish peroxidase-linked secondary antibodies and washed in TBST (3 periods, 5 min each time). Bands and blots were detected by enhanced chemiluminescence, while quantification was applied through laser scanning densitometry (GelPro Analyzer Software, GraphPad).

### 2.3. Determination of Intermediate Metabolism Enzyme Activities

Preparation of homogenates for assaying lactate dehydrogenase (L-LDH; E.C. 1.1.1.27.), citrate synthase (CS; E.C. 4.1.3.7.), and malate dehydrogenase (MDH; E.C. 1.1.1.37) was based on techniques by Driedzic & Almeida-Val [46], which are described in detail in Feidantsis et al. [47]. The enzymatic activities (*V*_max_) were determined spectrophotometrically at 18°C, and all assays were based on well-established protocols for fish tissues [48–50].

Specifically, for the analysis of LDH and MDH activities, samples were homogenized in a buffer containing 150 mM imidazole, 1 mM EDTA, 5 mM dithiothreitol (DTT), and 1% Triton X-100, pH 7.4. For CS activity, tissue samples were homogenized in a buffer containing 20 mM HEPES, 1 mM EDTA, with 1% Triton X-100, pH 7.4. To avoid loss of enzyme activity during sample preparation, procedures were performed on ice. Before analysis, homogenates were centrifuged at 13,000 x g for 10 min at 4°C. Maximum activity levels were determined spectrophotometerically at 18°C. L-LDH was assayed in a medium containing 0.15 mM NADH, 1 mM KCN, and 50 mM imidazole, pH 7.4. The reaction was initiated by adding 1 mM pyruvate. Malate dehydrogenase was assayed in a medium containing 0.15 mM NADH, 1 mM KCN, and 50 mM imidazole, pH 7.4. L-LDH and MDH enzyme activities were measured following the oxidation of NADH at 340 nm (*ε* = 6.22 mM^−1^ 1 cm^−1^). CS was assayed in a medium containing 0.4 mM acetyl CoA, 0.25 mM DTNB, and 75 mM Tris buffer, pH 8.0. The reaction was initiated by adding 0.5 mM oxaloacetate (OAA). CS enzyme activities were determined based on the reaction of free coenzyme A with DTNB (5, 5 V dithio-bis (2-nitrobenzoic acid) at 412 nm (*ε* = 13.6 mM^−1^ 1 cm^−1^).

Enzymatic activities were expressed as *μ*moles per minute per mg protein.

### 2.4. Determination of Activities of Antioxidant Enzymes

The frozen tissues were prepared for the measurement of antioxidant enzymes activity according to the protocol described in Salach [51]. Specifically, they were immediately homogenized in ice-cold phosphate buffer (50 mM, pH 7.4) 10% (w/v) using Omni international homogenizer (USA) at 22,000 rpm for 20 s each with 10 s intervals. The homogenate was centrifuged at 2,000 x g in cooling centrifuge at 4*°*C for 15 min, and the supernatant was saved. The supernatant was freeze-thawed thrice to completely disrupt mitochondria. Then the supernatant was again centrifuged at 6,000 x g in cooling centrifuge at 4°C for 15 min, and the yielded supernatant which contains the cytosolic and mitochondrial enzymes was saved for enzyme assays. The enzymatic activities (*V*_max_) were determined spectrophotometrically at 18°C, and all assays were based on well-established protocols for fish tissues [52–54].

Total superoxide dismutase (mitochondrial Mn- and cytosolic Cu/Zn-superoxide dismutase, SOD EC 1.15.1.1) activity was assayed by monitoring NADH oxidation. SOD activity was assayed by assessing the inhibition of NADH oxidation using *β*-mercaptoethanol in the presence of EDTA and Mn as a substrate. NADH solution was made fresh daily. The assays were run by adding to the cuvette sequentially 0.80 mL of 50 mM phosphate buffer (pH 7.4), 55 *μ*L EDTA/Mn solution of 100/50 mM, 40 *μ*L NADH solution of 7.5 mM and different volumes of tissue extract. The reaction was then initiated by adding 100 *μ*L 10 mM *β*-mercaptoethanol solution. The changes in the absorbance of NADH at 340 nM per min was followed (*ε* = 6.22 mM^−1^ 1 cm^−1^ at 340 nm). One unit of SOD activity is defined as the amount of tissue extract required to inhibit the rate of NADH oxidation of the control by 50% while the specific activity is expressed as units per mg protein.

Catalase (CAT, EC 1.11.1.6) activity was assayed as follows: 1 mL of 50 mM phosphate buffer (pH 7.4) and 10 *μ*L of tissue extract were added to the cuvette. The reaction was then initiated by the addition of 300 *μ*L of 30 mM H_2_O_2_ prepared by diluting 0.34 mL of 30% H_2_O_2_ to 100 mL of 50 mM phosphate buffer (pH 7.4). Catalase activity was determined following the changes in the absorbance of H_2_O_2_ at 240 nm (*ε* = 0.0394 mM^−1^ 1 cm^−1^ at 240 nm) and is expressed as *μ*mol per minute per mg protein.

Glutathione reductase (GR, EC 1.8.1.7) activity was assayed as follows: 1 mL of 50 mM sodium phosphate (pH 7.4) containing 2 mM EDTA and 0.15 mM NADPH and 10 *μ*L of tissue extract were added to the cuvette. The reaction was then initiated by the addition of 10 *μ*L of 1 mM GSSG. GR activities were determined following the changes in the absorbance of NADPH per min at 340 nm (*ε* = 6.22 mM^−1^ 1 cm^−1^).

Enzymatic activities were expressed as units per mg protein or *μ*mol per minute per mg protein.

### 2.5. Statistics

Changes in biochemical responses were tested for significance at the 5% level by using one-way Analysis of variance (ANOVA) (GraphPad Instat 3.0). Post-hoc comparisons were performed using the Bonferroni test. Values are presented as means ± S.D.

Changes in biochemical responses between examined species at the same TM diet were tested for significance at the 5% level by using t test (GraphPad Instat 3.0) followed by Kolmogorov–Smirnov test.

Principal components analysis (PCA) was conducted in the R package FactoMineR to assess patterns of possibly correlated variables and more specifically to detect how cellular stress responses, intermediate metabolism and antioxidant defence varied between temperatures and exposure time.

## 3. Results

### 3.1. Heat Shock Response (HSR)

**Figure 1:**
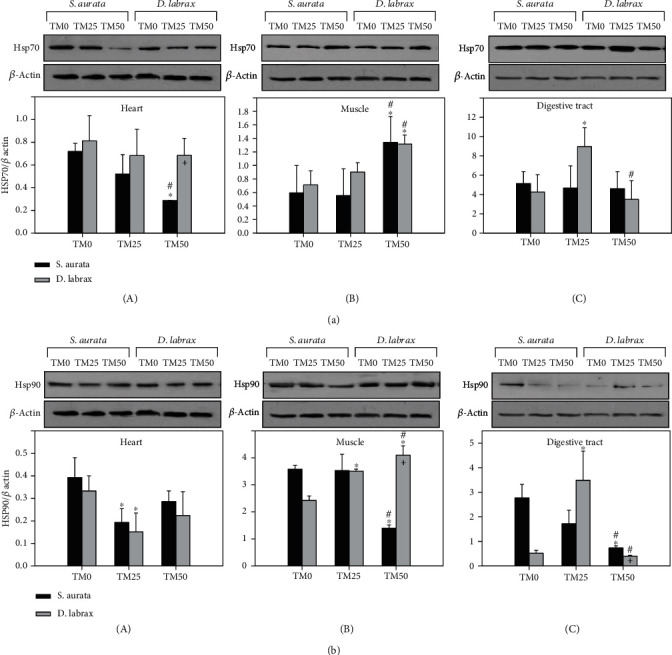
HSP70 (a) and HSP90 (b) in the heart (A), muscle (B), and digestive tract (C) of gilthead sea bream (*S. aurata*) and European sea bass (*D. labrax*) under the 0% (TM0), 25% (TM25), and 50% (TM50) *T. molitor* inclusion diet treatment. Representative immunoblots are shown and were quantified by laser scanning densitometry and plotted. Values represent means ± SD; *n* = 5 preparations from different animals. ∗ indicates *p* < 0.05 compared with 0%, ^#^ indicates *p* < 0.05 compared with 25%, and ^+^ indicates *p* < 0.05 between species at the same TM diet.

Regarding gilthead seabream, HSP70 levels in the heart (Figure 1(a)A) and muscle (Figure 1(a)B) exhibited differentiations only in response to the TM50 diet, while no differentiations were observed in the digestive tract (Figure 1(a)C). Specifically, in the heart (Figure 1(a)A), TM50 diet provoked a statistically significant decrease, while significantly induced levels were observed in the muscle (Figure 1(a)B) compared to both TM0 and TM25 diets. On the other hand, HSP70 expression in European sea bass presented no significant differentiations in the heart based on feeding regimes (Figure 1(a)A). However, European sea bass muscle exhibited a similar pattern to that of gilthead seabream muscle, with a statistically significant increase compared to both TM0 and TM25 diets (Figure 1(a)B). In contrast, HSP70 levels in the digestive tract of European sea bass statistically increased only in the TM25 diet, compared to both TM0 and TM50 diets (Figure 1(a)C). Regarding differences between species, HSP70 induction depicted higher levels in the heart of the European sea bass compared to the gilthead seabream in the TM50 regime (Figure 1(a)A).

HSP90 levels in the heart of both examined fish species exhibited a statistically significant decrease only in the TM25 diet, compared to both TM0 and TM50 diets (Figure 1(b)A). On the contrary, in the muscle (Figure 1(b)B) and digestive tract (Figure 1(b)C) of gilthead seabream, HSP90 levels decreased with increasing TM inclusion, with TM50 exhibiting statistically significant differences to both TM25 and TM50 diets. In the heart of European sea bass (Figure 1(b)A, HSP90 levels displayed a decrease only in the TM25 diet compared to the TM0 diet, while in the muscle (Figure 1(b)B), HSP90 levels increased with increasing diet regimes. Specifically, levels were significantly increased in the TM25 diet compared to TM0, and in the TM50 compared to both TM0 and TM25 diets. In European sea bass*'* digestive tract (Figure 1(b)C), only the TM25 diet significantly increased HSP90 levels compared to the other diet regimes. Species differences regarding HSP90 induction levels were evident only in the TM50 regime in the muscle and the digestive tract. While in the muscle, European sea bass displayed higher levels compared to the gilthead seabream (Figure 1(b)B), and the opposite pattern was depicted in the digestive tract (Figure 1(b)C).

### 3.2. MAPK Signalling

**Figure 2:**
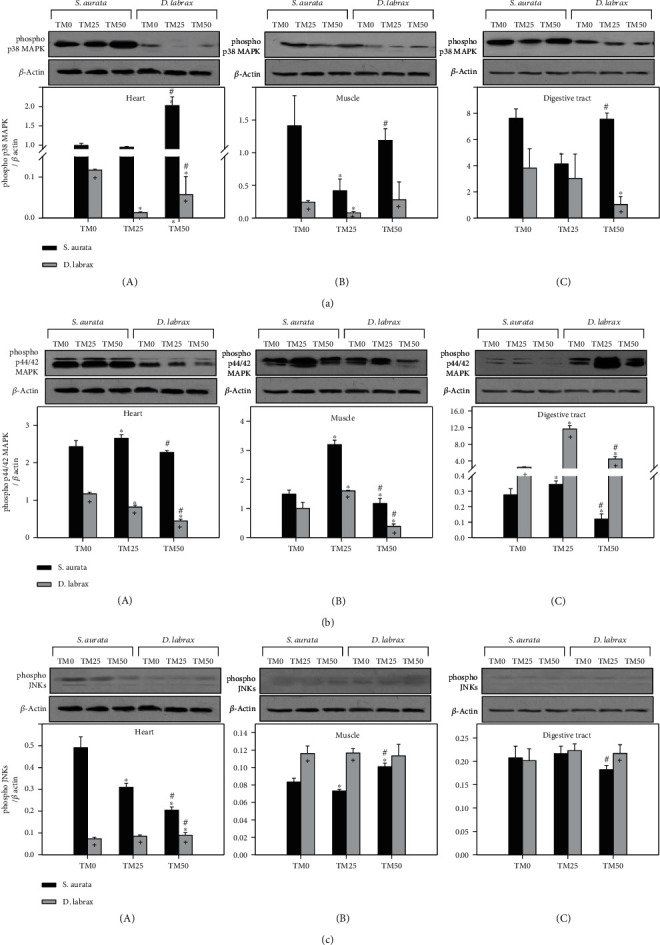
Phosphorylation of p38 MAPK (a), p44/42 MAPK (b), and JNKs (c) in the heart (A), muscle (B), and digestive tract (C) of gilthead sea bream (*S. aurata*) and European sea bass (*D. labrax*) under the 0% (TM0), 25% (TM25), and 50% (TM50) *T. molitor* inclusion diet treatment. Representative immunoblots are shown and were quantified by laser scanning densitometry and plotted. Values represent means ± SD; *n* = 5 preparations from different animals. ∗ indicates *p* < 0.05 compared with 0%, ^#^ indicates *p* < 0.05 compared with 25%, and ^+^ indicates *p* < 0.05 between species at the same TM diet.

The activation levels of p38 MAPK in the heart of gilthead seabream exhibited a statistically significant increase only in the TM50 diet (Figure 2(a)A). In gilthead seabream muscle (Figure 2(a)B) and digestive tract (Figure 2(a)C), p38 MAPK activation exhibited a similar pattern with a statistically significant decrease in the TM25 diet, while TM50 diet increased activation levels compared to TM0. Regarding European sea bass, TM25 diet provoked a p38 MAPK activation decrease in both heart (Figure 2(a)A) and muscle (Figure 2(a)B), while an increase in TM0 levels was observed in the TM50 diet. On the other hand, in the digestive tract (Figure 2(a)C), only the TM50 diet exhibited a significant decrease compared to TM0, while TM0 and TM25 exhibited no statistically significant differences.

Activation of p44/42 MAPK in the muscle and digestive tract of both species (Figure 2(b)B and Figure 2(b)C, respectively), and in the heart of gilthead seabream (Figure 2(b)A) exhibited a significant increase concerning the *ΤΜ*25 diet, while TM50 returned levels to that of TM0. However, in the heart of European sea bass (Figure 2(b)A), levels of p44/42 MAPK phosphorylation progressively decreased with increasing TM inclusion.

Regarding JNKs, gilthead seabream's heart exhibited a significant progressively decrease in its phosphorylation with increasing TM inclusion (Figure 2(c)A). In the muscle of the same species (Figure 2(c)B), TM25 diet provoked a statistically significant decrease, while the TM50 treatment statistically increased JNKs activation compared to both TM0 and TM25. In the digestive tract of gilthead seabream (Figure 2(c)C), the TM50 diet exhibited a significant decrease compared only to the T25 diet.

Regarding species differences, all MAPK members in the heart (Figures 2(a)A , 2(b)A, and 2(c)A) and p38 MAPK and p44/42 MAPK in the muscle (Figures 2(a)B and 2(b)A) exhibited lower phosphorylation levels in the European sea bass compared to the ones in the gilthead seabream. However, phosphorylation of JNKs in the muscle and digestive tract (Figures 2(c)B and 2(c)C) and phosphorylation of p44/42 MAPK in the digestive tract (Figures 2(b)C) exhibited increased levels in European sea bass compared to the ones in gilthead seabream in almost all feeding regimes.

### 3.3. Apoptosis

**Figure 3:**
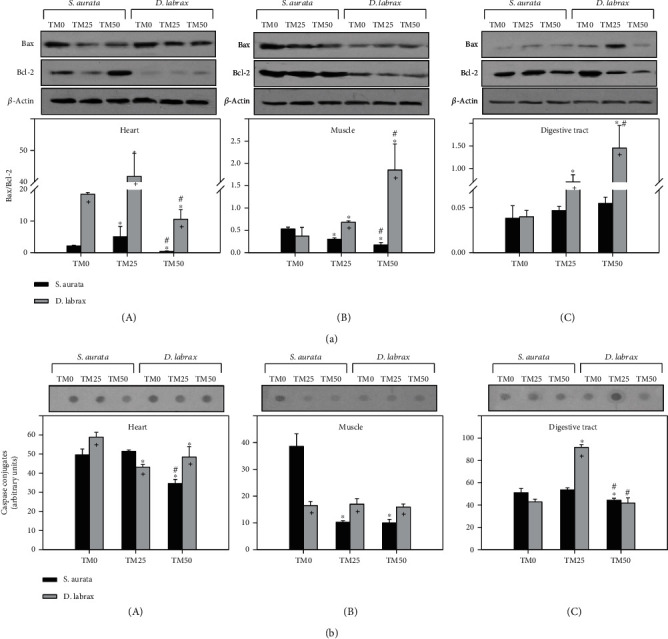
Bax/Bcl-2 ratio (a) and caspase conjugates (b) in the heart (A), muscle (B), and digestive tract (C) of gilthead sea bream (*S. aurata*) and European sea bass (*D. labrax*) under the 0% (TM0), 25% (TM25), and 50% (TM50) *T. molitor* inclusion diet treatment. Representative immunoblots are shown and were quantified by laser scanning densitometry and plotted. Values represent means ± SD; *n* = 5 preparations from different animals. ∗ indicates *p* < 0.05 compared with 0%, ^#^ indicates *p* < 0.05 compared with 25%, and ^+^ indicates *p* < 0.05 between species at the same TM diet.

Bax/Bcl-2 ratio exhibited no significant differences between *ΤΜ* inclusion diets in the digestive tract of gilthead seabream (Figure 3(a)C), while in the heart, TM25 diet resulted in a statistically significant increase of the ratio compared to TM0, and TM50 diet resulted in a decrease compared to both the TM25 and TM50 diets (Figure 3(a)A). The same pattern was also observed in the heart of European sea bass (Figure 3(a)A). Regarding Bax/Bcl-2 ratio in the muscle of gilthead seabream, a statistically significant progressive decrease in its levels with increasing TM inclusion was observed (Figure 3(a)B). On the other hand, the muscle (Figure 3(a)B) and digestive tract (Figure 3(a)C) of European sea bass presented a significant progressive increase in Bax/Bcl-2 ratio levels with increasing TM inclusion diets.

TM inclusion (TM25 and TM50) resulted in decreased caspases levels in all examined tissues of gilthead seabream. Specifically, while in the heart (Figure 3(b)C) and digestive tract (Figure 3(b)A), TM50 exhibited a statistically significant decrease compared to both TM0 and TM25 (which exhibited no difference between them), and in the muscle (Figure 3(b)B) of gilthead seabream both TM25 and TM50 (exhibiting no difference between them), they presented a statistically significant decrease compared to TM0. In European sea bass, a different pattern was observed, while in the muscle, no significant differences were observed between TM inclusion diets (Figure 3(b)B), and in the heart, both the TM25 and TM50 diets resulted in statistically significant decreased caspases levels compared to TM0 (Figure 3(b)A). An opposite pattern was observed in the digestive tract (Figure 3(b)C) with statistically significant increased caspases levels for TM25, while TM50 remained similar to TM0 levels.

Species differences were obvious in both Bax/Bcl-2 ratio and cleaved caspases levels in all examined tissues exhibiting higher levels in the European sea bass compared to the gilthead seabream in almost all feeding regimes (Figure 3).

### 3.4. Ubiquitination

**Figure 4:**
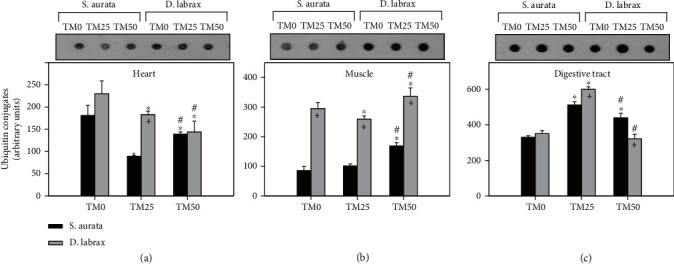
Ubiquitin conjugates in the heart (a), muscle (b), and digestive tract (c) of gilthead sea bream (*S. aurata*) and European sea bass (*D. labrax*) under the 0% (TM0), 25% (TM25), and 50% (TM50) *T. molitor* inclusion diet treatment. Representative immunoblots are shown and were quantified by laser scanning densitometry and plotted. Values represent means ± SD; *n* = 5 preparations from different animals. ∗ indicates *p* < 0.05 compared with 0%, ^#^ indicates *p* < 0.05 compared with 25%, and ^+^ indicates *p* < 0.05 between species at the same TM diet.

Ubiquitin conjugates levels in the heart of gilthead seabream exhibited a statistically significant decrease concerning the TM25 diet, while TM50 diet restored these levels, although still a statistically significant decrease compared to TM0 (Figure 4(a)). In the muscle of the same species, while both TM0 and TM25 diets presented no significant differences, TM50 diet provoked a statistically significant increase compared to both TM0 and TM25 (Figure 4(b)). In the heart of European sea bass (Figure 4(a)), the increasing TM inclusion resulted in a statistically significant progressive decrease, while in the muscle (Figure 4(b)), TM25 resulted in a significant decrease compared to TM0, and TM50 resulted in significantly increased ubiquitination levels compared to both TM0 and TM25 diets. The digestive tract of both examined species (Figure 4(c)) exhibited a similar pattern of ubiquitination with a statistically significant increase in TM25 diet compared to TM0, while the TM50 diet significantly decreased the ubiquitination levels, compared to TM25.

While in the heart and the digestive tract of the European sea bass, ubiquitin conjugates levels were higher only in the TM25 regime, and in the muscle, these levels were higher in all feeding regimes compared to the gilthead seabream (Figure 4).

### 3.5. Autophagy

**Figure 5:**
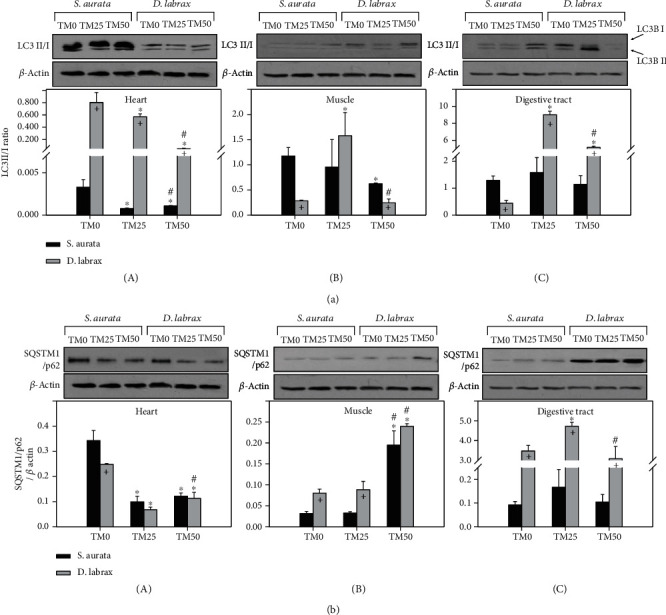
LC3BII/LC3BI ratio (a) and SQSTM1/p62 (b) in the heart (A), muscle (B), and digestive tract (C) of gilthead sea bream (*S. aurata*) and European sea bass (*D. labrax*) under the 0% (TM0), 25% (TM25), and 50% (TM50) *T. molitor* inclusion diet treatment. Representative immunoblots are shown and were quantified by laser scanning densitometry and plotted. Values represent means ± SD; *n* = 5 preparations from different animals. ∗ indicates *p* < 0.05 compared with 0%, ^#^ indicates *p* < 0.05 compared with 25%, and ^+^ indicates *p* < 0.05 between species at the same TM diet.

In the heart of gilthead seabream, the LC3 II/I ratio significantly decreased in both TM25 and TM50 diets compared to TM0. However, TM50 diet significantly increased LC3 II/I ratio compared to TM25 diet (Figure 5(a)A). In the muscle of the same species, only TM50 diet significantly decreased the LC3 II/I ratio compared to TM0, while the TM25 diet had no effect (Figure 5(a)B). Contrary to the above, the digestive tract exhibited no variations between TM inclusion regimes (Figure 5(a)C). Regarding European sea bass, in the heart LC3 II/I ratio significantly and progressively decreased with increasing TM inclusion (Figure 5(a)A). On the other hand, in the muscle and the digestive tract of European sea bass, an increase was noted in the TM25 diet, while the TM50 diet provoked a decrease in the observed LC3 II/I ratio (Figures 5(a)B and 5(a)C).

SQSTM1/p62 levels in the heart of both examined species significantly decreased in the TM25 and TM50 diets compared to the TM0 diet. However, in European sea bass, the TM50 diet provoked a statistically significant increase compared to TM25 (Figure 5(b)A). Concerning the muscle of both gilthead seabream and European sea bass, only the TM50 diet resulted in a statistically significant increase in SQSTM1/p62 levels compared to TM0 and TM25, while the latter diets exhibited no significant variations (Figure 5(b)B). In the digestive tract of European sea bass, TM25 diet provoked a significant increase in SQSTM1/p62 levels, while TM50 diet restored this protein's levels to these of TM0. On the contrary, the digestive tract of gilthead seabream exhibited no significant variations between the TM feeding regimes (Figure 5(b)C).

While autophagy in the heart of European sea bass was more evident compared to the gilthead seabream (Figures 5(a)A and 5(b)A), the opposite pattern was observed in the muscle (Figures 5(a)B and 5(b)B). Interestingly, in the digestive tract, both LC3 II/I and SQSTM1/p62 levels were higher in the European sea bass compared to the gilthead seabream (Figure 5(a)C and 5(b)C).

### 3.6. Antioxidant Defence

**Figure 6:**
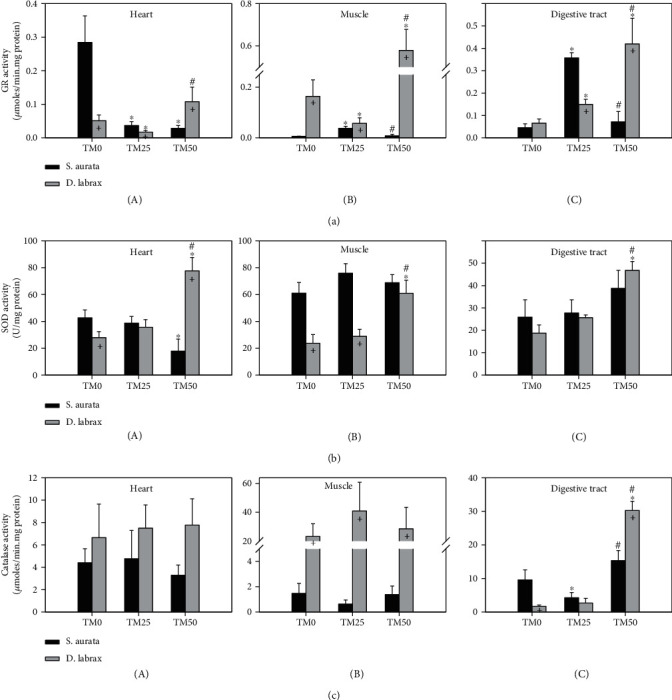
Activities of glutathione reductase (GR) (a), superoxide dismutase (SOD) (b), and catalase (CAT) (c) in the heart (A), muscle (B), and digestive tract (C) of gilthead sea bream (*S. aurata*) and European sea bass (*D. labrax*) under the 0% (TM0), 25% (TM25), and 50% (TM50) *T. molitor* inclusion diet treatment. Values represent means ± SD; *n* = 5 preparations from different animals. ∗ indicates *p* < 0.05 compared with 0%, ^#^ indicates *p* < 0.05 compared with 25%, and ^+^ indicates *p* < 0.05 between species at the same TM diet.

In the heart of gilthead seabream, both TM25 and TM50 diets resulted in a similar decrease in GR activity levels compared to TM0 (Figure 6(a)A). However, both the muscle and digestive tract exhibited a similar pattern with TM25 diet statistically increasing GR activity levels compared to TM0 and TM50, decreasing them to the levels of TM0 (Figures 6(a)B and 6(a)C, respectively). In European sea bass, TM25 resulted in a decrease and TM50 resulted in an increase of GR activity levels in both the heart and the muscle of this species (Figures 6(a)A and 6(a)B, respectively). However, in the digestive tract, the increase in TM inclusion resulted in a progressively significant increase of GR activity levels (Figure 6(a)C).

In the muscle and the digestive tract of gilthead seabream, TM inclusion regimes resulted in no changes in SOD activity levels (Figures 6(b)B and 6(b)C, respectively). In the heart, however, only the TM50 diet caused a statistically significant decrease compared to TM0 (Figure 6(b)A. Regarding European sea bass, TM50 caused a statistically significant increase of SOD activity in both the heart and muscle, while no changes were observed between TM0 and TM25 diets (Figures 6(b)A and 6(b)B, respectively). In the digestive tract, the increasing TM inclusion progressively elevated SOD activity levels (Figure 6(b)C).

Catalase activity levels remained stable in the heart and the muscle of both fish species under the effect of TM inclusion regimes (Figures 6(c)A and 6(c)B, respectively). In the digestive tract of gilthead seabream, the TM25 diet statistically decreased SOD activity levels compared to TM0, but the TM50 diet increased SOD activity to levels similar to that of TM0 (Figure 6(c)C). In the same tissue in European sea bass, only the TM50 diet resulted in a statistically significant increase compared to both TM0 and TM25 (Figure 6(c)C).

Regarding species differences, GR and SOD activity levels in the heart were lower in the TM0 regime in the European sea bass compared to the gilthead seabream. However, the opposite pattern was exhibited in the TM50 regime in the same tissue (Figures 6(a)A and 6(b)A). In the muscle, GR and catalase activity levels were higher in all feeding regimes in the European sea bass compared to the gilthead seabream (Figures 6(a)B and 6(c)B), while the opposite was observed regarding SOD activity (Figure 6(b)B. GR and catalase activity levels in the digestive tract displayed increased levels in the European sea bass compared to the gilthead seabream only in the TM50 regime, while the opposite was observed in the lower feeding regimes (Figures 6(a)C and 6(c)C).

### 3.7. Intermediate Metabolism

**Figure 7:**
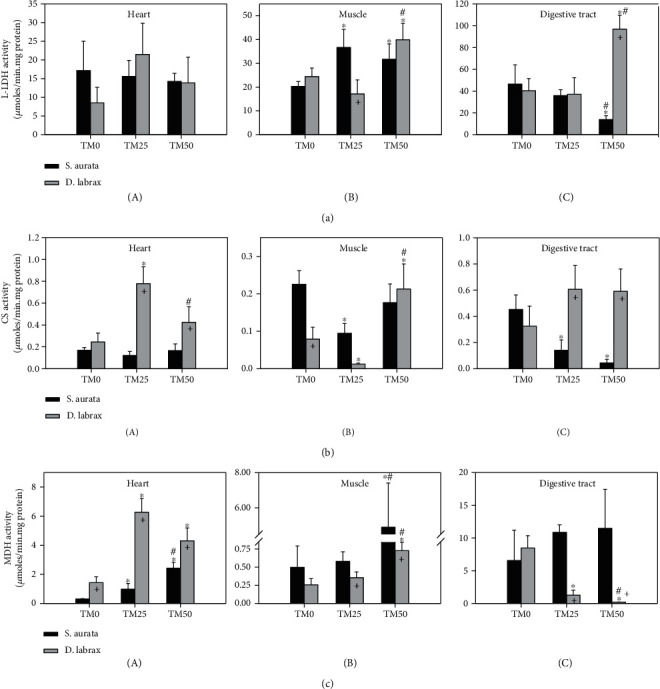
Activities of lactate dehydrogenase (L-LDH) (a), citrate synthetase (CS) (b), and malate dehydrogenase (MDH) (c) in the heart (A), muscle (B), and digestive tract (C) of gilthead sea bream (*S. aurata*) and European sea bass (*D. labrax*) under the 0% (TM0), 25% (TM25), and 50% (TM50) *T. molitor* inclusion diet treatment. Values represent means ± SD; *n* = 5 preparations from different animals. ∗ indicates *p* < 0.05 compared with 0%, ^#^ indicates *p* < 0.05 compared with 25%, and ^+^ indicates *p* < 0.05 between species at the same TM diet.

L-LDH activity levels in the heart of both species remained unchanged despite the differences in TM inclusion (Figure 7(a)A). However, in the muscle of gilthead seabream, both TM25 and TM50 provoked increased L-LDH activity compared to TM0, but no statistically significant differences were observed between them (Figure 7(a)B). In the digestive tract of the same species, only the TM50 diet provoked a decrease in L-LDH activity levels, which was statistically significant both to TM0 and TM25 (Figure 7(a)C). The muscle (Figure 7(a)B) and the digestive tract (Figure 7(a)C) of European sea bass exhibited a similar pattern; although the TM25 diet provoked no changes compared to TM0, the TM50 diet statistically increased L-LDH activity compared to both TM0 and TM25.

CS activity levels remained unchanged in the heart of gilthead seabream under the different TM regimes (Figure 7(b)A). In the muscle (Figure 7(b)B), only the TM25 diet resulted in decreased CS activity levels compared to TM0 and TM50, while in the digestive tract (Figure 7(b)C), both TM25 and TM50 significantly decreased CS activity, with no statistical differences between the two regimes. Although the TM inclusion regimes did not cause any changes in the CS activity in European sea bass digestive tract (Figure 7(b)C), TM25 diet provoked a statistically significant increase of CS activity in the heart, while TM50 reduced these levels compared to TM25 (Figure 7(b)A). In the muscle, a different pattern was observed as TM25 diet statistically reduced CS activity levels compared to TM0, and TM50 caused a statistically significant increase compared to both TM0 and TM25 (Figure 7(b)B).

Regarding the heart of gilthead seabream, increasing TM inclusion significantly decreased MDH activity levels in a progressive manner (Figure 7(a)A). In the muscle of the same species, only the TM50 diet provoked a statistically significant increase in MDH activity levels compared to both TM0 and TM25 (Figure 7(a)B), while in the digestive tract, MDH activity increased in a progressive manner with increasing TM inclusion (Figure 7(a)C). In the heart of European sea bass, both TM25 and TM50 diets resulted in a statistically significant increase of MDH activity (Figure 7(c)A). In the muscle of the same species, the TM50 diet resulted in a significant increase compared to both TM0 and TM25 (Figure 7(c)B). However, in the digestive tract, the increase in TM inclusion led to a progressively significant decrease in MDH activity (Figure 7(c)C).

Regarding species differences, L-LDH activity levels in the digestive tract (Figure 7(a)C), CS activity levels in the heart and the digestive tract (Figures 7(b)A and 7(b)C), and MDH activity levels in the heart (Figure 7(c)A) depicted increased levels (mostly in the TM25 and TM50 regimes) in the European sea bass compared to the gilthead seabream. However, all enzymes of intermediate metabolism examined herein exhibited decreased enzymatic activity levels in the European sea bass compared to the gilthead seabream (Figures 7(a)B, 7(b)B, and 7(c)B).

### 3.8. Multivariate Analysis

**Figure 8:**
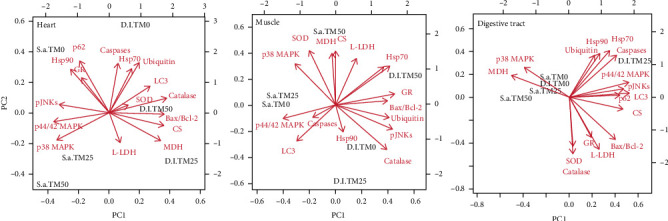
Variable correlations of heart, liver, and digestive tract with each of the first two principal components (PCs) in the multivariate analysis. The PCA was generated from the complete biochemical dataset. Parameters with red vector arrows were included as PCA construction predictors (S.a.: *S. aurata*; D.l.: *D. labrax*).

In the heart of both examined species, PC1 explained 44.54% of the variance. The physiological variables that were positively correlated with PC1 scores were Bax/Bcl-2 ratio, LC3 II/I ratio, CS, MDH, SOD, and catalase, forming clusters with the TM50 feeding regime in European sea bass. The physiological variables that were positively correlated with scores on PC2, which explained 26.83% of the variance, were HSP70 and HSP90, caspases, ubiquitin, SQSTM1/p62, and GR, forming clusters mainly with the TM0 feeding regime in gilthead seabream. The cumulative value of PC1 and PC2 was 71.37% (Figure 8).

In the liver of fish species, PC1 explained 38.28% of the variance. The physiological variables that were positively correlated with PC1 scores were *Η*sp70, phospho JNKs, Bax/Bcl-2 ratio, ubiquitin, SQSTM1/p62, GR, and catalase, which mainly formed clusters with the TM50 feeding regime in European sea bass. The physiological variables that were positively correlated with PC2, which explained 26.82% of the variance, were phospho p38 MAPK, L-LDH, CS, MDH, and SOD, mainly forming clusters with the TM50 feeding regime in gilthead seabream. The cumulative value of PC1 and PC2 was 65.1% (Figure 8).

In the digestive tract of both examined species, PC1 explained 46.53% of the variance. The variables that were positively correlated with PC1 scores were phospho p44/42 MAPK, phospho JNKs, Bax/Bcl-2 ratio, LC3 II/I ratio, SQSTM1/p62, L-LDH, CS, and GR, mainly forming clusters with the TM25 and TM50 feeding regimes in European sea bass. The variables that were positively correlated with PC2 (explaining 32.59% of the variance) were HSP70, HSP90, phospho p38 MAPK, caspases, ubiquitin, and MDH, which mainly formed clusters with the TM0 feeding regime in European sea bass and all feeding regimes in gilthead seabream. The cumulative value of PC1 and PC2 was 79.12% (Figure 8).

### 3.9. Aggregated Results

**Figure 9:**
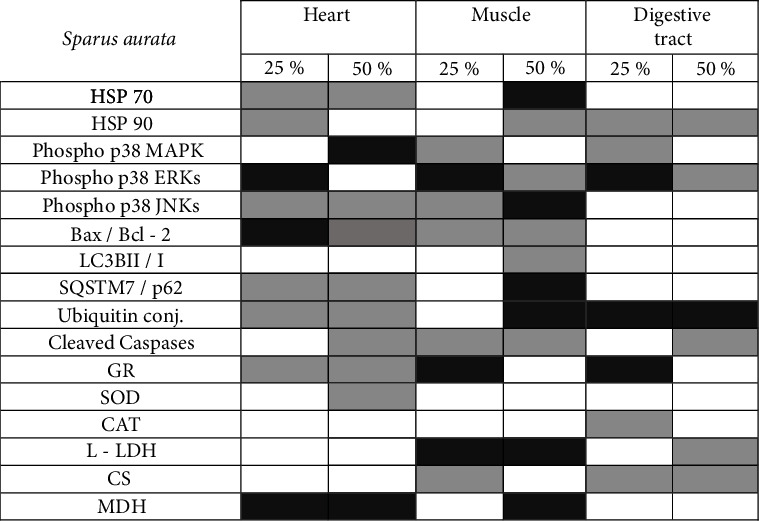
Effect of partial substitution (25 and 50%) of fish meal with insect meal (IM) on the four examined tissues (heart, liver, muscle, and digestive tract) of the gilthead sea bream (*Sparus aurata*). Dark grey indicates increase, light grey indicates decrease and white indicates no change compared to the fish meal–control (0% substitution).

**Figure 10:**
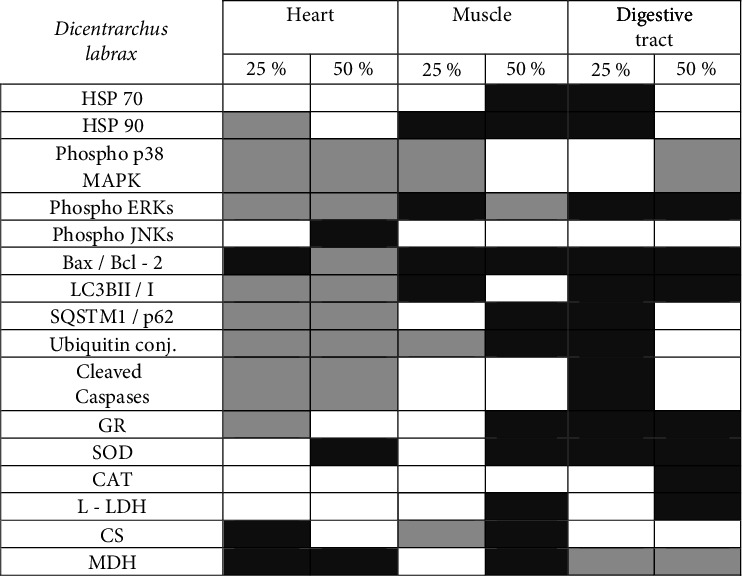
Effect of partial substitution (25 and 50%) of fish meal with insect meal (IM) on the four examined tissues (heart, liver, muscle, and digestive tract) of the sea bass (*Dicentrarchus labrax*). Dark grey indicates increase, light grey indicates decrease, and white indicates no change compared to the fish meal–control (0% substitution).

Figures 9 and 10 depict the increase, decrease, or no variation regarding the overall expression of examined proteins and the enzymatic activities in the heart, muscle, and digestive tract of both fish species.

## 4. Discussion

Although research regarding the fish meal supplementation with plant-based diets and their effect on fish biology and physiology has been conducted recently [e.g., [39, 55]], literature linking supplementation of fish meal (especially with insect meal), as well as molecular and biochemical pathways in fish is, to our knowledge, scarce. Our previous research has provided insight into the effect of TM larvae meal inclusion on the hepatic proteome and apoptotic and autophagic processes in three farmed fish species, including gilthead seabream and European sea bass [56]. However, due to the fact that fish biochemical processes are characterized by intense tissue specificity [57, 58] and the need for sustainable aquaculture practices [59], more research regarding fish molecular and metabolic pathways in response to fish meal substitutes is of high importance.

### 4.1. Stress Responses

In the present study, the most abrupt observed increases in HSP70 and HSP90 expression were primarily due to TM50 inclusion in the muscle of both fish species and in their digestive tract under the TM25 regime. These induced HSP levels could be attributed to both the initiation of cellular stress responses due to fish feed composition changes [29, 30, 60], as well as the increase of HSPs' quantity, necessary for protein metabolism, due to increased influx of free amino acids from TM inclusion [61, 62]. Similarly, the relative gene expression of Hsp70 in hepatopancreas of common carp *Cyprinus carpio* (Linnaeus, 1758) significantly increased when 75% or more FM protein was replaced with defatted black soldier fly *Hermetia illucens* (Linnaeus, 1758) larvae [63]. Similar results were obtained in the liver of rainbow trout under the effect of graded dietary inclusion level of full-fat black soldier fly prepupae meal. Specifically, 50% inclusion resulted in an increased Hsp70 gene expression in the liver of the aforementioned species [64]. Likewise, Ji et al. [65] have shown that 70% replacement of dietary fish meal with silkworm pupae meal is able to induce the relative Hsp70 gene expression in the intestine of *C. carpio*.

However, decreases or no changes in HSPs' expression levels were observed in the heart of both gilthead sea bream and European sea bass. Moreover, substitution of fish meal with 66% and 100% black soldier fly larvae meal has been reported to decrease the Hsp70 and Hsp27 gene expression in the head kidney leukocytes of Atlantic salmon *Salmo salar* (Linnaeus, 1758) [66]. In other fish species, such as the false *percula* clownfish *Amphiprion ocellaris* (Cuvier, 1830), replacement of fish meal with 25%, 50%, and 75% black soldier fly larvae meal has shown no significant differences in the expression of several genes, including Hsp70 [67]. Physiological responses to dietary inclusion of insect meal have also been investigated in mammals. Specifically, in 5-week-old male crossbred pigs [Piétrain × (German Landrace × German Edelschwein)], the inclusion of yellow mealworm larvae at 5% and 10% levels has led to no changes in the gene expression of hepatic Hsp70 [68].

In addition, an identical pattern of differential phosphorylation levels was also exhibited regarding the MAPKs family members examined herein. Variations in MAPK phosphorylation depended both on fish species as well as tissue examined. This pattern could be attributed to the fact that MAPKs' activation is involved in the regulation of HSPs in fish tissues [69]. Following the decrease of Hsps' gene expression, 66% and 100% black soldier fly larvae meal has also resulted in a decrease of p38 MAPK gene expression in Atlantic salmon's head kidney leukocytes [66]. However, knowledge regarding the MAPK signalling pathway in fish under the effect of insect meal feed remains understudied. On the contrary, partial fish meal supplementation with plant-based meal in fish species of high commercial value has revealed a tissue- and inclusion percentage-specific manner regarding both MAPK pathway and HSR [55].

### 4.2. Apoptosis & Autophagy

The apoptotic responses in both examined fish species in the present study showcased a similar pattern to the above differential. In general, apoptosis seems to be induced in all tissues under the effect of both TM25 and TM50. However, in the muscle of gilthead seabream, the gradual increase of TM meal inclusion resulted in a parallel gradual decrease of apoptosis [56]. Inclusion of the same insect meal at 25%, 50%, and 75% levels has resulted in increased activity of caspase 3, as well as apoptosis in the 75% group in the liver of largemouth bass *Micropterus salmoides* (Lacépède, 1802). Similarly, casp3, casp9, casp8, and casp10 relative mRNA levels were upregulated in both 50% and 75% TM inclusion groups [70]. On the contrary, 5% and 10% yellow mealworm larvae inclusion in feed exhibited no variations in Bax gene expression in the liver of growing pigs [68]. Thus, despite the fact that Ringseis et al. [68] support that their feeding regimes provoked no cell death responses in their mammalian model, the present results, as well as those by Su et al. [70] lead to the opposite conclusion.

Although apoptosis usually goes together with autophagy, experimental or environmental conditions, as well as differences in species, tissues, and stimuli may favour each side in this tug of war. Specifically, although in general autophagy, it can prevent apoptosis by recycling unnecessary or dysfunctional cellular components [71, 72], and under prolonged and/or intense stress conditions, autophagy may serve as an apoptotic process, finally leading to cell death [73, 74]. Although apoptosis seems to prevail over the autophagic process in the heart of both fish species in the present study, both of these processes are triggered in the muscle and digestive tract. The latter has also been observed in the liver of the gilthead seabream when fed with TM regimes [56]. Probably, in these tissues, the effect of TM substitution acts as a strong stress stimulant, since in both the European sea bass and the rainbow trout, hepatic autophagy was able to suppress apoptosis [56]. However, from the results obtained in the present study, it is not clear whether cells in these tissues will undergo or survive apoptosis, or how the latter is linked to autophagy and to the subsequent cellular and organismal homeostasis.

### 4.3. Antioxidant Defence

Although the term “dietary oxidative stress” has been employed to describe an imbalance between prooxidants and antioxidants, which derives from nutrients supply insufficiency in fish [75], it has also been pointed out that changes in food availability or food quality during an organism's lifetime may impact its ability to produce antioxidants, and thus defend against oxidative stress [76]. However, the underlying mechanisms linking food with oxidative stress and/or antioxidant defence in fish remain to be elucidated. The results of the present study exhibit an increased antioxidant capacity in the European sea bass rather than the gilthead sea bream due to TM inclusion. Specifically, while in the European sea bass, most of the antioxidant enzymes increased their activities, particularly under the TM50 feeding regime, and in the gilthead sea bream, the most abrupt increases due to TM inclusion (TM25) were observed in the muscle and digestive tract.

In contrast to the present results, increasing TM meal inclusion (TM25 → TM50) results in a parallel increase of SOD and GR activity in proximal intestine of rainbow trout [77]. Similarly, black soldier fly meal dietary inclusion at 25% and 50% levels in rainbow trout has resulted to a differential pattern of antioxidant capacity in the liver and kidney of this species, generally indicating the shielding of *H. illucens* meals-mediated oxidative process [78]. Despite the fact that the same insect meal inclusion has resulted in no lipid peroxidation in the African catfish *Clarias gariepinus* (Burchell, 1822), catalase activity was found to be increased, indicating an elevated antioxidant defence [79]. Similar to the above, defatted black soldier fly meal has proven to boost the antioxidant status of pearl gentian grouper (*Epinephelus fuscoguttatus* × *E. lanceolatus*) [80] and Jian carp, by higher catalase activity [63]. Similarly, defatted black soldier fly meal has resulted in tissue-specific, but in general, it increased lipid peroxidation as well as antioxidant defence efficiency in the liver and the kidney of the Siberian sturgeon *Acipenser baerii* (Brandt, 1869) juveniles [81].

On the other hand, replacement of fish meal with increasing black soldier fly larvae meal has resulted in decrease in the gene expression of enzymes involved in the antioxidant defence of Atlantic salmon head kidney leukocytes [66]. However, in false percula clownfish, fish meal replacement with black soldier fly resulted in no changes in GR gene expression levels [67]. Similar results were obtained in the liver of rainbow trout under the effect of graded dietary inclusion levels of full-fat black soldier fly prepupae meal. In specific, the 50% inclusion level, but not the 25%, resulted in no alterations in GR gene expression in the liver of the aforementioned species [64]. Moreover, yellow mealworm larvae meal in pigs has also led to no changes in SOD and catalase activity in the liver and gastrocnemius muscle [68].

Although oxidative stress has been reported to implicate in the initiation of apoptosis [82] and autophagy [83], it is not clear whether this kind of stress triggers cellular responses or if the latter are initiated by specific food ingredients or nutrient deficiencies. Nevertheless, it is obvious from the conducted PCA analysis that cell death and autophagy parameters mainly form clusters with antioxidant enzymes. However, since fish oil levels were lower in the TM inclusion regimes and thus less susceptible to peroxidation processes, it can be assumed that the fish studied herein exhibited antioxidant capacity originating from the antioxidants contained in their diet. The latter is also supported by the metabolic activation and (in combination with an increase in oxygen consumption) initiation of oxidative stress [75, 84].

### 4.4. Intermediate Metabolism

Since insects represent an innovative ingredient rich in high-quality proteins and other beneficial nutrients (fats, minerals and vitamins) [16, 85–87], the main attribute of yellow mealworm in its larval stage is that it has a high protein content (47% to 60% crude protein), presenting itself as a potential alternative protein source in animal feed [88–91]. Therefore, the high in crude protein and well-balanced essential amino acids' profile of insect meal are expected to fuel the citric acid cycle, since the cycle itself provides precursors of certain amino acids. From the obtained results, it is apparent that in gilthead seabream, the heart is mainly fueled by the MDH activity. This highlights the significance of oxaloacetate, which is fueled both by carbohydrates and amino acids, in the citric acid cycle. In the heart of European sea bass, both MDH and CS increased under the TM feeding regime, which also exhibits the importance of several nutrients including lipids in the metabolism of this tissue. However, in the muscle, L-LDH (together with MDH) seems to prevail, highlighting the involvement of glucose in insect-fed fish. In the digestive tract of the gilthead sea bream, MDH seems to provide the available energy through oxaloacetate, since CS and L-LDH are downregulated, while European sea bass seems to depend more on glucose metabolism under TM treatment. It has been demonstrated that defatted yellow mealworm larvae meal in the European sea bass diet differentially activates genes involved in fatty acid metabolism [92].

Contrary to the above, replacement of fish meal with black soldier fly larvae meal has resulted in no variations in the expression of genes involved in mitochondrial metabolism (COX2 and LOX5) in Atlantic salmon head kidney leukocytes [66]. Likewise, replacement of fish meal with 30% and 50% black soldier fly and with 50% yellow mealworm resulted in no significant changes in enzymes of the intermediary metabolism (such as FPBase, PK and G6PDH) in the liver of gilthead seabream [93].

Hence, the above underline the fact that metabolic processes are extremely versatile and depend on many factors, such as fish species, tissue fuel preferenda, feed regime, age, and experimental protocol. Given that most of the ongoing research examines the effects of insect meal inclusion on growth performance and meat quality, the necessity of studies focusing on the metabolic aspects of dietary insects is indisputable and necessary in order to elucidate how intermediate metabolism contributes to individual welfare.

## 5. Conclusion

The antioxidant defence and metabolic, cellular, and molecular pathways under the influence of yellow mealworm inclusion in the diet of two economically important species (gilthead seabream and European sea bass) exhibited versatile variations, which are species, tissue, and TM inclusion oriented. In contrast to gilthead seabream, European sea bass appeared to be more susceptible to dietary yellow mealworm, where induction of both HSPs and the apoptotic and autophagic machinery was more prominent. Both fish species' heart seems to also rely on lipids compared to muscle and the digestive tract. However, the differential biochemical responses observed herein between the two fish species may be attributed to deviations of insect-meal diets from the natural nutritional requirements of each species. Even though an accurate conclusion about TM effect could not be obtained, due to the tissue- and species-specific signaling, the present study could provide the basis for future research regarding feed stimuli and fish physiological performance.

## Figures and Tables

**Table 1:** Composition of the experimental diets.

Composition (g/kg)	*S. aurata*			*D. labrax*		
	0%	25%	50%	0%	25%	50%
Fish meal	500.0	330.0	130.0	700.0	450.0	200.0
*T. molitor* meal	0.0	250.0	500.0	0.0	250.0	500.0
Wheat flour	—	—	—	92.0	90.0	80.0
Wheat bran	—	—	—	55.0	40.0	25.0
Corn gluten	150.0	125.0	130.0	0.0	28.0	0.0
Wheat gluten	—	—	—	50.0	75.0	150.0
Starch (gelatinized, D500)	180.0	170.0	150.0	0.0	0.0	12.0
Fish oil	140.0	95.0	60.0	90.0	54.0	20.0
Trace metals supplement	10.0	10.0	10.0	—	—	—
Vitamins supplement	10.0	10.0	10.0	—	—	—
Binder	10.0	10.0	10.0	—	—	—
Methionine	**—**	**—**	**—**	6.0	6.0	6.0
Lysine	**—**	**—**	**—**	3.0	3.0	3.0
Choline	**—**	**—**	**—**	1.5	1.5	1.5
Vitamins and trace metals mixture (premix)	**—**	**—**	**—**	2.5	2.5	2.5
Total	1000.0	1000.0	1000.0	1000.0	1000.0	1000.0

## Data Availability

Data are available on request.
